# Cultural Differences in Tweeting about Drinking Across the US

**DOI:** 10.3390/ijerph17041125

**Published:** 2020-02-11

**Authors:** Salvatore Giorgi, David B. Yaden, Johannes C. Eichstaedt, Robert D. Ashford, Anneke E.K. Buffone, H. Andrew Schwartz, Lyle H. Ungar, Brenda Curtis

**Affiliations:** 1 Computer and Information Science Department, University of Pennsylvania, Philadelphia, PA 19104, USA; sgiorgi@sas.upenn.edu (S.G.); ungar@cis.upenn.edu (L.H.U.); 2 National Institutes of Health, National Institute on Drug Abuse, Bethesda, MD 20892, USA; 3 Department of Psychology, University of Pennsylvania, Philadelphia, PA 19104, USA; dyaden@sas.upenn.edu (D.B.Y.);; 4 Department of Psychology & Institute for Human-Centered Artificial Intelligence, Stanford University, Stanford, CA 94305, USA; johannes@jeichstaedt.com; 5 Substance Use Disorders Institute, University of the Sciences, Philadelphia, PA 19104, USA; rashford@mail.usciences.edu; 6 Department of Computer Science, Stony Brook University, Stony Brook, NY 11794, USA; has@cs.stonybrook.edu

**Keywords:** excessive drinking, social media, Twitter, natural language processing, American Communities Project

## Abstract

Excessive alcohol use in the US contributes to over 88,000 deaths per year and costs over $250 billion annually. While previous studies have shown that excessive alcohol use can be detected from general patterns of social media engagement, we characterized how drinking-specific language varies across regions and cultures in the US. From a database of 38 billion public tweets, we selected those mentioning “drunk”, found the words and phrases distinctive of drinking posts, and then clustered these into topics and sets of semantically related words. We identified geolocated “drunk” tweets and correlated their language with the prevalence of self-reported excessive alcohol consumption (Behavioral Risk Factor Surveillance System; BRFSS). We then identified linguistic markers associated with excessive drinking in different regions and cultural communities as identified by the American Community Project. “Drunk” tweet frequency (of the 3.3 million geolocated “drunk” tweets) correlated with excessive alcohol consumption at both the county and state levels (*r* = 0.26 and 0.45, respectively, *p* < 0.01). Topic analyses revealed that excessive alcohol consumption was most correlated with references to drinking with friends (*r* = 0.20), family (*r* = 0.15), and driving under the influence (*r* = 0.14). Using the American Community Project classification, we found a number of cultural markers of drinking: religious communities had a high frequency of anti-drunk driving tweets, Hispanic centers discussed family members drinking, and college towns discussed sexual behavior. This study shows that Twitter can be used to explore the specific sociocultural contexts in which excessive alcohol use occurs within particular regions and communities. These findings can inform more targeted public health messaging and help to better understand cultural determinants of substance abuse.

## 1. Introduction

Excessive alcohol consumption, including binge and heavy drinking, is responsible for approximately 88,000 deaths per year in the US, making it the third leading preventable cause of death and a major public health concern [1,2,3]. Binge drinking, generally defined as having five or more drinks for males and having four or more drinks for females in about 2 hours [4], was reported by 26.4% of people ages 18 or older in 2017 [5]. Binge drinking is associated with adverse health effects such as unintentional injuries (e.g., falls, motor vehicle crashes), alcohol poisoning, interpersonal violence (e.g., homicide, assaults, domestic violence), risky sexual behaviors, and suicide [6]. Additionally, excessive alcohol consumption in the US costs over $250 billion annually, due to the loss in workplace productivity, health care expenses, law enforcement expenses, and motor vehicle crashes [1]. The individual, societal, and economic costs associated with binge drinking has led to repeated calls to monitor alcohol use and its associated adverse health effects as well as to implement public health interventions that reduce alcohol-attributed risks. 

A key element of public health alcohol interventions is the monitoring of binge and heavy drinking [7,8]. Traditionally, monitoring involves large surveys (such as the Centers for Disease Control and Prevention’s Behavioral Risk Factor Surveillance System; BRFSS) which track trends over time and across specific populations such as metropolitan statistical areas. However, such expensive survey efforts with a limited number of questions cannot shed light on the different cultural practices that lead to excessive drinking, even though such information is necessary to target public health media campaigns [9,10,11]. Specifically, given the variability in alcohol consumption and associated risks across the United States, it is important to have a granular appreciation for the ways in which different populations within the US engage in excessive alcohol use. Effectively targeted public health communication involves delivering different messages based on predetermined segmentations (e.g., demographic and socioeconomic characteristics), yielding tailored communication which is more likely to persuade individuals [12].

Social media analysis provides an untapped resource for monitoring alcohol consumption at the population level. Social media provides autobiographical language expressing thoughts, emotions, and behaviors, and with the application of machine learning models to such language data, individual psychometric assessments (such as estimates of personality [13]) can be derived. Social media data provide real-time and cost-efficient monitoring of large fractions of populations which in principle can extend across a rich variety of psychological characteristics [14,15,16]. This data source could provide prevention program planners with the ability to gather information that can be used to tailor public health messaging.

Popular social media platforms in the United States include Facebook, Twitter, Instagram, and Snapchat. Twitter is a free social media platform that allows users to send and receive “tweets” (i.e., short messages limited to 280 characters). As of the last quarter in 2017, Twitter averaged 330 million monthly active users, creating an estimated 500 million tweets per day [17]. Given the widespread use and activity on Twitter as well as the availability of public content at the user level, it has been used in multiple health surveillance studies. For example, Twitter data have been used to track influenza symptoms, estimate alcohol sales volume, and measure depression, HIV prevalence, and heart disease mortality [18,19,20,21,22,23].

Twitter language data have been used to predict alcohol consumption rates at the county level [24]. Curtis et al. [24] used natural language analysis to associate Twitter language content to rates of alcohol consumption at the county level. Findings revealed that Twitter language data captured cross-sectional patterns of excessive alcohol consumption beyond that of sociodemographic factors (e.g., age, gender, race, income, education). Twitter data have also been used to examine drinking themes and sentiment, showing that drinking tweets contained positive sentiment and as well as themes of wanting, needing, and planning [25]. Social media platforms are also sources of information about substance use patterns in particular sub-populations. For example, the results of a meta-analysis that examined the relationship between young adults’ alcohol-related social media engagement and their drinking behavior revealed a relationship between alcohol-related social media engagement and alcohol-related problems [14].

In this study, we examined the feasibility of using Twitter to monitor binge drinking with a focus on regional and cultural differences. Specifically, we addressed the following questions: (1) Do Twitter messages expressing language indicative of excessive alcohol use correlate with county-level alcohol consumption rates? (2) What are the contents of these binge drinking-related tweets? (3) What insights can we gain from examining the regional and cultural variations in the language of these tweets? Finally, (4) can linguistic features, such as pronoun use and valence, help to characterize drunk content within communities? Our aim was to examine the efficacy of social media language analysis as an emerging tool for public health monitoring and intervention.

## 2. Materials and Methods

### 2.1. Data

#### 2.1.1. Excessive Alcohol Consumption Data

The BRFSS is a population-based cross-sectional phone health survey of US adults aged ≥ 18 years conducted by state health departments with funding and technical assistance provided by the Centers for Disease Control and Prevention. From the BRFSS (2006–2012), we obtained the prevalence of self-reported binge drinking and heavy drinking (for which county-level estimates had previously been derived; *N* = 2192; [7]). Excessive alcohol consumption was defined as having more than two drinks per day on average (for men) or more than one drink per day on average (for women) or having five or more drinks during a single occasion (for men) or four or more drinks during a single occasion (for women). 

#### 2.1.2. Drinking Keyword Filtering

In order to identify tweets related to excessive drinking, we started with 46 drinking-related, unambiguous keywords, such as “hangover”, “tailgate”, “vodka”, and “wasted”, introduced in Cavazos-Rehg et al. [25] in addition to our initial keyword “drunk” for a total of 47 keywords (see the full list of keywords in Appendix A, Table A1). These keywords were introduced by Cavazos-Rehg et al. [25] in order to study general drinking-related discussions on Twitter, in terms of sentiment, theme, and source. Since the keywords were used to identify general patterns of drinking, we also used them to identify drinking-related tweets in order to build our “drunk tweet” data set. From our larger Twitter data set (described below), we collected a random sample of roughly 153,000 tweets containing at least one least one of the 47 keywords. Next, for each tweet we created 47 binary indicators for each of our keywords (1 if the tweet contained the keyword, 0 otherwise) and correlated all pairs of binary indicators to identify keyword patterns in the drunk tweets. The results showed that most drinking keywords were relatively rare and did not correlate with other keywords, including “drunk”. The three most common words “sober” (*N* = 4120), “bar” (*N* = 2218), and “ale” (*N* = 2124). Additionally, most keywords did not co-occur within the same tweet. Therefore, we chose to limit our data set to tweets which contained the word “drunk”, to focus on tweets describing the act of drinking itself, rather than its effects (e.g., “hungover” and “hangover”).

#### 2.1.3. Twitter Data

**Figure 1:**
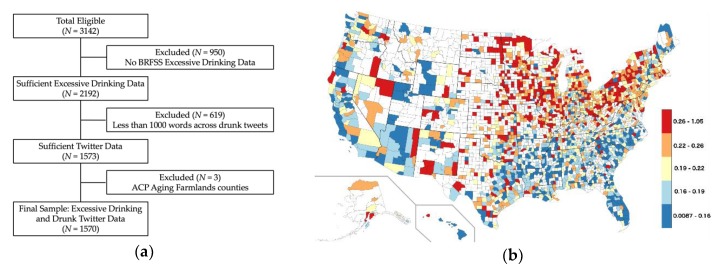
Data description: (**a**) inclusion criteria for this study; (**b**) map of drunk tweet frequency (quintiles, red = high; blue = low).

A random 10% of Twitter data were collected between June 23, 2009 to April 17, 2014, augmented with a 1% sample from April 17, 2014 to February 5, 2015 [26,27]. This resulted in approximately 37.6 billion tweets. The Tweets were then filtered so that the word “drunk” appeared in the tweet (we removed any tweets that contained the phrase “drunk in love” due to the popular song title). This resulted in a set of roughly 24.9 million tweets. All non-English tweets were removed using the Python package langid [28]. After language filtering, 19.3 million tweets remained which were then mapped to US counties. Using the geolocation methods described in Reference [29], we used self-reported location information in user profiles and latitude/longitude coordinates attached to tweets to map tweets to US counties and county equivalents (henceforth “counties”). This resulted in 3.3 million “drunk tweets” spread over 3095 counties. Finally, we limited our analysis to counties with at least 1000 words within the drunk tweets, for a total of *N* = 1573 counties in our final data set. Summary results are given in Figure 1a and a US map of drunk tweet frequency in Figure 1b.

#### 2.1.4. American Communities Project

In addition to county- and state-level analyses, we also looked at 15 community types identified by the American Communities Project (ACP) [30]. The ACP is a county-level clustering based on 36 demographic, socio-economic, and cultural indicators including population density, income, race, and religious affiliation and developed by George Washington University’s School of Media and Public Affairs. Sample communities include Big Cities, College Towns, Hispanic Centers, and Rural Middle America. Note that this county clustering does not depend on spatial proximity, for example, the Big Cities cluster contains counties across the US which contain large metropolitan areas such as Los Angeles and Philadelphia. We argue that this clustering scheme gives more culturally coherent interpretation to expressions of drinking on social media than either counties or states. Additionally, restricting the analysis to a small number of distinct community types allows for the possibility of public health officials developing more culturally tailored messaging and interventions than if our analyses were restricted to states (which are often socio-demographically heterogeneous) or counties (for which there are over 3000 units). We limited our analysis to ACP communities for which at least 25% of the counties met our 1000 “drunk” word threshold. This resulted in 14 ACP communities across *N* = 1570 counties (with Aging Farmlands dropped due to the fact insufficient data).

### 2.2. Topic Modeling

The set of English-filtered “drunk” tweets (19.3 million) were used to create a set of 100 “drunk” topics using latent Dirichlet allocation (LDA) [31]. To create the drunk topics, we (1) tokenized each tweet (i.e., broke up each tweet into words and word phrases), (2) identified those words and phrases most associated with drunk tweets, (3) filtered the tweets to only contain the most discriminative words (i.e., removed words not significantly associated with drunk tweets), and (4) ran the LDA algorithm over the full set of filtered drunk tweets. Each step is described in detail below.

In their natural form, tweets exist as strings of text which need to be broken up into words whose frequency can be recorded. Early versions of this process (“tokenizing”) used white spaces to split a sentence into words, but we used a more modern version designed to handle social media language data (which may include, for example, “:)!” to be broken up into “:)” and “!”) [32]. This allowed us to describe the frequency of every word in a tweet as the fraction of the total number of words (“tokens”) in that tweet (i.e., Step 1 in the preceding paragraph). We also encoded if a word was used at all in a given tweet (as a “binary” feature: 1 if a token is present in the message, 0 if otherwise). We further recorded the relative frequency of phrases (such as “happy birthday”) which we detected by observing them to be more frequently co-occurring words than chance would suggest based on the frequency of “happy” and “birthday”.

In Step 2, we found the words and word phrases associated with “drunk” tweets. We randomly sampled 1 million random tweets from our “drunk” set and gathered 1 million random “non-drunk” messages (i.e., messages without the word “drunk”) from roughly the same time span. We then calculated a weighted log odds ratio, using an informative Dirichlet prior to estimate the difference in frequency of a word across two corpora (i.e., drunk tweets and non-drunk tweets) [33,34]. This method uses the z score of the log odds ratio in order to control the variance of a given word’s frequency, while the prior shrinks the word frequency towards known frequencies from a large background corpus.

In the third step, we filtered each of the 19.3 million drunk messages to only contain 5000 tokens most associated with “drunk” tweets (i.e., any word *not* within the top 5000 significantly correlated tokens was removed from the tweet). Using these 19.3 million filtered messages, we created 100 topics using LDA. The LDA topics were estimated using Gibbs sampling [35] with the MALLET software package [36]. For an extended description of this process, see Schwartz et al. [13].

Finally, in Step 4, we calculated county-level topic loadings for each of the 100 drunk topics using:

(1) $$P\left(topic|county\right)=\underset{\left\{token\in topic\right\}}{\sum }P\left(topic|token\right)\times P\left(token|county\right).$$

Here *P*(*topic*|*token*), the probability of a topic given a token, was derived via the LDA process and *P*(*token*|*county*) was estimated using the relative frequency of the token in the county. These topic frequencies were then used as independent variables in the statistical analysis.

### 2.3. Statistical Methods

#### 2.3.1. Drunk Tweeting and Excessive Drinking

We first explored the relationship between Twitter language data identified as “drunk tweets” and excessive drinking by correlating the frequency of drunk tweets (i.e., the number of drunk tweets divided by the total number of tweets within a county) with the BRFSS measure of excessive drinking. We did this at the county, state, and ACP levels (both the state and ACP level variables were calculated as county-level averages).

#### 2.3.2. Differential Language Analysis

We use differential language analysis (DLA) to identify (1) language characterizing counties higher and lower in excessive drinking and (2) drunk language most associated with individual ACP communities [13]. For the former (1), we individually regressed (via a least squares linear regression) each language feature (i.e., county-level topic frequencies for each of the 100 drunk topics) against the BRFSS excessive drinking measure. For the latter (2), we attempted to identify regional trends in drunk tweets by identifying topics most associated with each ACP community. To do this, we created a county-level dummy outcome for each of the 14 ACP communities in our sample (1 if the county is in the ACP community, 0 if otherwise). We considered the association of all 100 drunk topics with all 14 ACP communities using Cohen’s d which quantifies the differences in means among subsamples of counties in units of pooled standard deviations. Additionally, for each topic we computed the *p*-value associated with its coefficient in a logistic regression. For both (1) and (2), we applied a Benjamini–Hochberg correction to the significance threshold (*p* < 0.05) of the false discovery rate for multiple comparisons [37].

#### 2.3.3. Self versus Other Drinking

We looked at references to self and other drinking in our drunk tweets. For each county, we calculated the relative frequency of the word “I” (self) in our drunk tweets as well the frequency of “he”, “she”, and “they” (other). We then standardized (i.e., mean centered and normalized) both the “self” and “other” scores. 

#### 2.3.4. Sentiment

Finally, we examined the relationship between sentiment and personal pronouns. To measure personal pronoun use, we used the “personal pronoun” dictionary in the Linguistic Inquiry Word Count (LIWC) which contains 93 distinct pronouns [38]. This method simply counts the number of occurrences of each pronoun within each ACP community. To measure positive sentiment, we used the National Resource Council (NRC) Hashtag Sentiment Lexicon which is designed to estimate tweet sentiment in a robust fashion [39]. This lexicon differs from LIWC, in that each word in the lexica contains a weight and thus gives us a weighted sum of all positive sentiment words occurring in each ACP community.

## 3. Results

### 3.1. Community Correlations with Excessive Drinking

**Figure 2:**
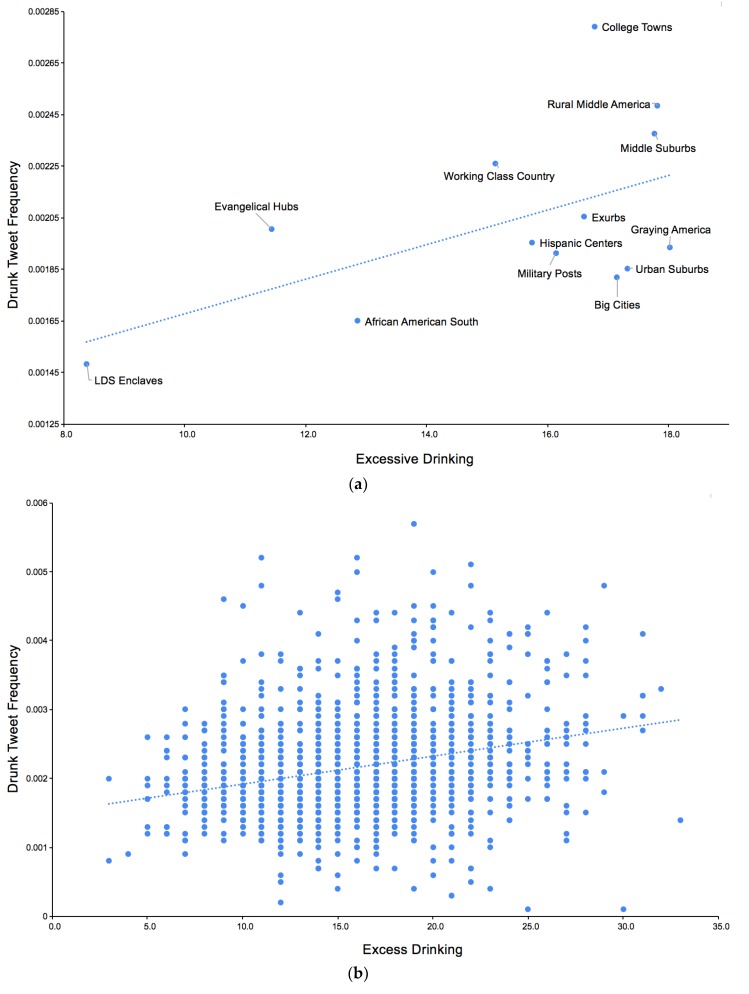
Frequency of drinking tweets versus excessive drinking levels: (**a**) ACP classification (county averages) and (**b**) county level.

The frequency with which people tweet the word “drunk” (as a percentage of all tweets) is moderately correlated with excess drinking at both the county (Pearson’s *r* = 0.26) and state (Pearson’s *r* = 0.45) level as shown in Table 1. Across the 14 categories of counties determined by the American Communities Project (ACP), we observed a relationship between excessive drinking rates and drunk tweet frequency with trending significance (Pearson’s *r* = 0.55, *p* = 0.053), seen in the linear relationship in Figure 2. Based on the ACP classification, on the one hand, we observed that areas with stronger religious identification (LDS Enclaves (Latter-Day Saints; Mormon) and Evangelical Hubs) as well as the African American South were both lowest in excessive drinking and drunk tweeting.

On the other hand, we observed that College Towns were near the top in excessive drinking and were distinguished from all other categories of communities by the extent to which they tweet about drinking. After College Towns, Rural Middle America and Middle Suburbs tweeted the most about drinking. These two communities are predominantly white (91% in Rural Middle America and 85% in Middle Suburbs) and low income.

### 3.2. Differential Language Analysis

**Figure 3:**
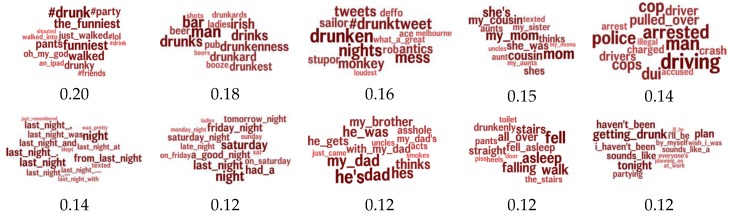
Drunk topics most positively correlated with county-level excess drinking. Reported Pearson’s *r*, *p* < 0.05 after Benjamini–Hochberg correction.

Figure 3 shows the drunk topics most associated with high levels of excessive drinking at the county level. These topics include a number of hashtags related to drinking and partying with friends (“#fun”, “#shots”, “#lastnight”, “#drunksanta”), two topics related to drinking with family (“my cousins”, “my mom”, “my aunts”, “with my dad”, “my brother”) as well as a DUI-related topic (“got pulled” arrested”, “pleads”) and a topic mentioning falling both asleep and down the stairs.

**Figure 4:**
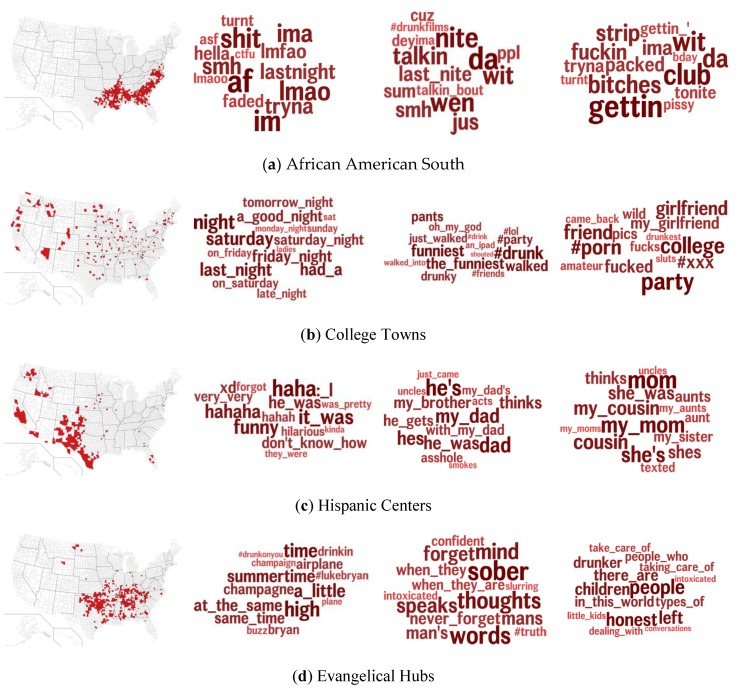
Maps and drunk topics most associated with four ACP communities: (**a**) African American South, (**b**) College Towns, (**c**) Hispanic Centers, and (**d**) Evangelical Hubs. Left most plots indicate the counties (red) within each ACP community.

In terms of the ACP, Figure 4 presents the topics associated with four ACP communities: (a) African American South, (b) College Towns, (c) Hispanic Centers, and (d) Evangelical Hubs. These particular communities and topics were selected from the top five most correlated topics (i.e., highest Cohen’s *d*). These communities and topics provide insight into the kind of social contexts in which drinking is discussed.

African American communities, besides showing higher use of African American English (“tryna”, “da”, “ima”), also mentioned (night) clubs [40]. College Towns discussed drinking on the weekends (“friday night”, “Saturday”, “a good night”) and drinking humor (“the funniest”, “drunky”, “oh my god”) as well as sex-related topics (“pics”, “#porn”, “party”, “fucks”). In Hispanic centers, the social context of drinking was often foregrounded (“my dad”, “my mom”, “my cousin”). Evangelical Hubs used particular terms to reference drinking (“buzz”, “drinkin”, “high”) but also to reference sobriety (“sober”, “intoxicated”, “never forget”) and responsibility (“take care of”, “dealing with”, “little kids”).

### 3.3. Self versus Other Drinking

Tweeting about drinking may both reference one’s own behaviors (“I am drunk and fell asleep”) as opposed to references to others’ behaviors (“He was drunk and fell down the stairs”). To differentiate these kinds of references, we compared mentions of the word “I” (self; first person) against mentions of “he”, “she” and “they” (other; third person) among drinking-related tweets in Table 2. LDS Enclaves, Rural Middle America, and College Towns all have an overall pattern of impersonal tweeting about drinking (using fewer pronouns both about the self and others). The African American South marks the contrary case, where tweeting about drinking was personal with higher reference to both self and other drinking.

### 3.4. Sentiment

**Figure 5:**
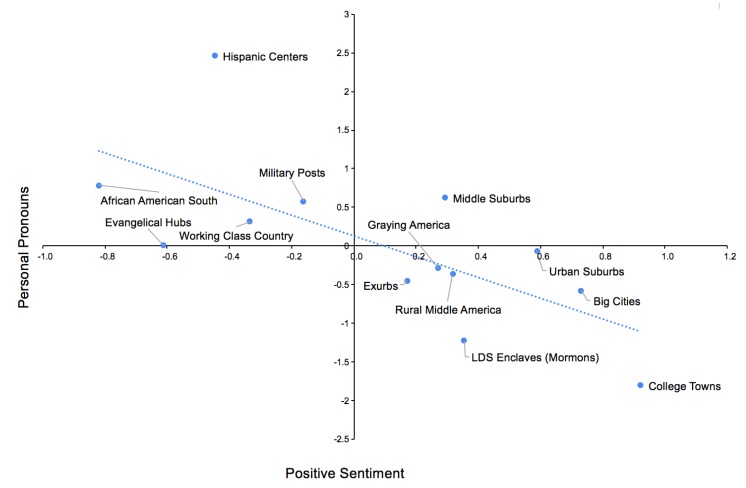
Scatter plot of personal (versus impersonal) tweeting against positive sentiment across the 14 ACP community types. Sentiment is based on the NRC positive sentiment model. Both dimensions were standardized.

How does the personal/impersonal nature about tweeting relate to the way drinking is perceived across types of communities? Figure 5 shows how the personal nature of tweeting about drinking (measured as the relative frequency of personal pronouns) relates to the sentiment of the tweets. We generally observed that positive tweets about drinking tend to be more impersonal and thus may reference general practices or cultural norms more so than experiences of oneself or others; this was particularly true in College Towns. Inversely, tweets containing personal pronouns tended to be more negative in valence with Hispanic Centers sharing by far the most personal content.

## 4. Discussion

In this study, we found that Twitter can be used as a lens into regional and community context around excessive alcohol use. Communities differed both in *how much* they tweet about drinking and *how* they tweet about drinking. This suggests that Twitter can be used to estimate trends in health behaviors across counties and that a similar methodology can be applied to comorbid conditions such as substance use, depression, and anxiety. In light of our research questions, *how much* addresses our first question: do Twitter messages expressing language indicative of excessive alcohol use correlate with county-level alcohol consumption rates. The *how* addresses the remaining three questions: What are the contents of binge drinking-related tweets? Can pronoun use and valence help characterize drunk tweet content? Finally, what insights can we gain from examining the regional and cultural variations in the language of these tweets?

First, we answer our initial research question in the affirmative: we found that the frequency of “drunk tweeting” accurately captured the trend in the rates of excessive drinking captured by phone surveys administered by the CDC. This finding dovetails with previous studies that showed that simple keyword searches on Twitter predict county-level outcomes (i.e., flu keywords predict influenza-like illness statistics and “drunk” predicts alcohol sales) [18]. This finding underscores the promise for social media platforms to be used as a public health tool for monitoring health-related behaviors. Additionally, the results suggest that religious communities are both the least likely to drink excessively and Tweet about drinking, while university and white, low-income communities see the most tweeting about drinking. The LDS Enclaves finding in particular provides a methods check, as the Mormon religion does not permit drinking, so it should be—and is—at the bottom of this ranking.

Second, in regard to *how* communities Tweet about drinking, we found two main dimensions of distinction addressing our research questions on the content of drunk-related tweets and how this content varies regionally and culturally. Topic analyses revealed that drinking may get discussed either in the form of celebratory endorsements of the cultural practice of drinking (e.g., in College Towns) or in a cautionary manner (e.g., by Mormon communities). Communities also differed in how much discussions around drinking take the form of *personal* behaviors (of both oneself and others) versus how much they are referenced *impersonally* as cultural practices. Between these two dimensions, we found a trend suggesting that disclosures with personal pronouns tended to be more negative, and more impersonal references to cultural norms and practices tended to be more positive. This was mostly the case in College Towns. This suggests that drinking may be perceived positively as a shared practice and negatively “when it happened to me last night”. While the interpretation of these differences of language use around drinking requires additional research to confirm, they represent interesting and potentially important avenues to explore the relevance to public health intervention and messaging to these communities. For example, changing the view of drinking as a social practice towards the negative may prove fruitful, perhaps akin to the successful messaging around smoking which made a “cool” practice a distasteful one.

There are also suggestions of strong regional differences in mentions of family (e.g., aunts, cousins, and parents) with Hispanic Centers having a number of significant family-related topics. We note that Hispanic Centers were highest in self-drinking and, therefore, these communities were talking about both family drinking and self-drinking.

A notable standout in terms of communities was College Towns, which tweeted about drinking at a much higher frequency than the excessive drinking rate would suggest. We suspect this reflects a younger and more social media friendly population than most of the other ACP communities. Sexual themes were also associated with College Towns. We note that both porn (#xxx, #porn, sluts) and significant others (my girlfriend, friend) were contained within the same topic. Previous studies have found both substance abuse and pornography consumption as risk factors for male sexual aggression on college campuses [41].

Using publicly available Twitter data as a surveillance system has been used across a number of health-related outcomes including excessive drinking [24]. A similar surveillance study examined hashtags co-occurring in tweets mentioning e-cigarettes while also looking at rates of tobacco-related tweets across the US. [42]. This study contextualized e-cigarette tweets in terms of both co-occurring hashtags and across *spatially* connected regions (i.e., Mid-Atlantic, Southwest, and the West Coast). The present study differs in that drunk tweets were contextualized across *culturally* connected regions, namely, the American Communities Project classification—counties grouped via socio-demographic measures as opposed to groups of adjacent counties.

This study was limited in several ways. These analyses were conducted using aggregated Twitter data and regional drinking figures drawn from large-scale surveys conducted by the CDC. These findings should be replicated using individual-level data with linked self-reports and language. Similar studies have shown associations between self-reported behavior and tweet content. In particular, Unger et al. [43] showed that posting positive tweets about tobacco had a significant association with tobacco use within the past month.

There is also the potential for a selection bias. It may be that only certain types of people tweet about being drunk. However, people do tweet a lot about smoking marijuana, even when and where it is illegal [44]. It may be that there are fewer social taboos on Twitter in regard to discussing substance use than in “polite conversation” thus allowing users a relative sense of freedom to post what they wish. Here again, individual-level data with self-reported drinking behavior and personal language will help to determine whether this is an issue.

Twitter bots can be a source of noise as well as false behaviors and attitudes. Previous studies on social media and e-cigarettes have shown that bots are more likely to reference new e-cigarette products than human Twitter users [40]. One can imagine similar patterns in drinking-related tweets—bots might be more likely to tweet about a new alcoholic beverage or drinking-related social event. Thus, future studies should examine similar relationships between social media bots and mentions of alcohol consumption.

The words, phrases, and linguistic themes that emerge from natural language processing are predictive of outcomes of interest, yet care must be taken when interpreting the results, and for final validity, they should be tested in confirmatory studies that test the identified associations as hypotheses.

## 5. Conclusions

Excessive alcohol use was reflected and detectable in patterns of social media language. Regions and communities differed both in terms of the quantity and content of social media posts about drinking. We found that tweets about being drunk were predictive of different “styles” of excessive drinking behavior across types of communities derived from demographic and socio-economic indicators in the American Communities Project. The particular words, phrases, and linguistic themes most associated with particular regions and communities can provide insight into sociocultural alcohol use contexts and may help to shape more personalized public health messages and interventions to these populations.

## Figures and Tables

**Table 1:** Correlations between drunk tweet frequency and excess drinking at the county, state, and American Communities Project levels. Reported Pearson’s *r* with 95% confidence intervals in square brackets.

Spatial Unit	*N*	Correlation with Excessive Drinking
County	1573	0.26 [0.21, 0.31] (*p* < 0.001)
State	46	0.45 [0.18, 0.72] (*p* = 0.002)
American Communities Project (ACP)	14	0.55 [−0.007, 1.103] (*p* = 0.053)

**Table 2:** Standardized (z scored) relative frequency of “I*”* and “he/she/they*”* within drunk tweets.

	Self	Other
Hispanic Centers	1.90	0.18
African American South	1.53	2.55
Middle Suburbs	1.00	0.18
Military Posts	0.97	0.38
Urban Suburbs	0.73	0.18
Big Cities	0.31	0.77
Native American Lands	−0.59	0.38
LDS Enclaves	−0.61	−1.20
Graying America	−0.69	0.58
Rural Middle America	−0.74	−1.59
College Towns	−0.75	−1.20
Exurbs	−0.81	−0.41
Evangelical Hubs	−0.97	−0.01
Working Class Country	−1.26	−0.80

## Appendix A

In Table A1 we list all of our drinking keywords.

**Table A1:** Full list of drinking keywords.

alcohol	bottle	hangover	pub
alcoholics	bottles	happy hour	shot(s)
alcoholism	brewery	hungover	sober
ale	champagne	lager	tailgate
bar	ciroc	liquor	tailgating
beer	cocktail	lounge	tequila
beer goggles	cocktails	margarita(s)	tipsy
beers	drank	pint	vodka
booze	drink	pints	wasted
boozey	drinking	pregame	whiskey
boozy	gin	pregaming	wine
